# No species is an island: testing the effects of biotic interactions on models of avian niche occupation

**DOI:** 10.1002/ece3.1387

**Published:** 2015-01-17

**Authors:** Federico Morelli, Piotr Tryjanowski

**Affiliations:** 1 Faculty of Biological Sciences, University of Zielona GoraProf. Z. Szafrana St. 1, Zielona Gora, 65-516, Poland; 2 Faculty of Environmental Sciences, Department of Applied Geoinformatics and Spatial Planning, Czech University of Life Sciences PragueKamýcká 129, Prague 6, 165 00, Czech Republic; 3 Institute of Zoology, Poznan University of Life SciencesWojska Polskiego 71 C, Poznań, 60-625, Poland

**Keywords:** Avian niche, biotic interactions, red-backed shrike, species association, species distribution models

## Abstract

Traditionally, the niche of a species is described as a hypothetical 3D space, constituted by well-known biotic interactions (e.g. predation, competition, trophic relationships, resource–consumer interactions, etc.) and various abiotic environmental factors. Species distribution models (SDMs), also called “niche models” and often used to predict wildlife distribution at landscape scale, are typically constructed using abiotic factors with biotic interactions generally been ignored. Here, we compared the goodness of fit of SDMs for red-backed shrike *Lanius collurio* in farmlands of Western Poland, using both the classical approach (modeled only on environmental variables) and the approach which included also other potentially associated bird species. The potential associations among species were derived from the relevant ecological literature and by a correlation matrix of occurrences. Our findings highlight the importance of including heterospecific interactions in improving our understanding of niche occupation for bird species. We suggest that suite of measures currently used to quantify realized species niches could be improved by also considering the occurrence of certain associated species. Then, an hypothetical “species 1” can use the occurrence of a successfully established individual of “species 2” as indicator or “trace” of the location of available suitable habitat to breed. We hypothesize this kind of biotic interaction as the “heterospecific trace effect” (HTE): an interaction based on the availability and use of “public information” provided by individuals from different species. Finally, we discuss about the incomes of biotic interactions for enhancing the predictive capacities on species distribution models.

## Introduction

**Figure 1:**
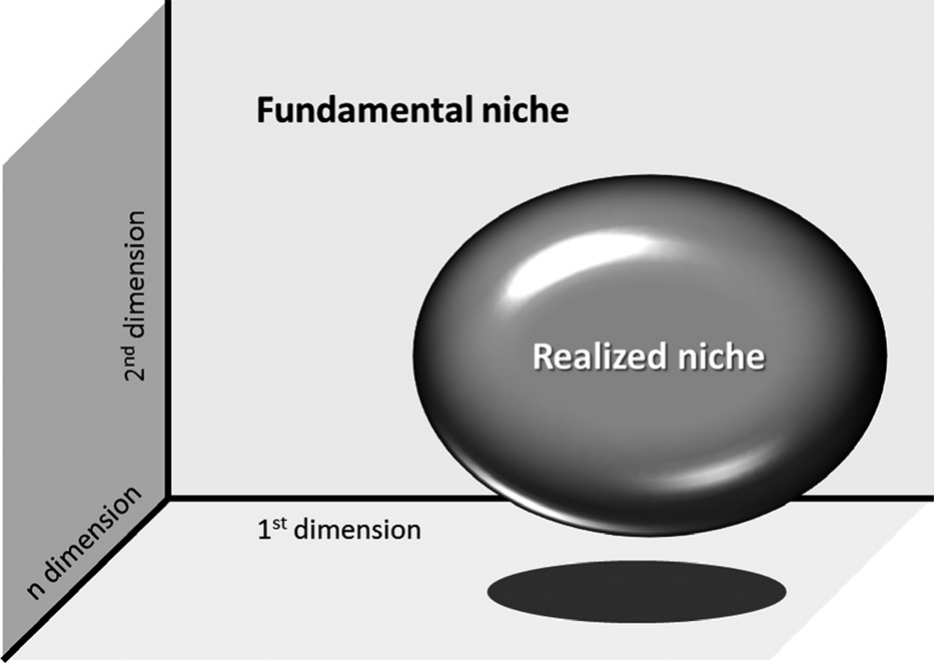
Schematic representation of fundamental and realized niche of an hypothetical species, in a n-dimensional space. Each dimension or axis represents the range of some environmental condition or resource that is required by the species (Hutchinson 1957).

Niche theory describes species’ distribution in terms of an hypothetical 3D space (Colwell and Rangel 2009). Hutchinson (1957) defined the niche as the volume in multidimensional hyperspace in which species can maintain a viable population. The entire hypervolume within which an organism can potentially exist describes its fundamental niche, whereas the portion of the fundamental niche that a species actually occupies (e.g. due to competitive exclusion) defines its realized niche (Fig. 1). The realized or fundamental niche can be viewed in terms of either the Eltonian niche (the functional attributes of a species and its corresponding trophic position: Elton 1927) or the Grinnellian niche (the response of a species to the abiotic and biotic environment: Grinnell 1917). However, most studies concerning species niches have been focused on environmental characteristics of sites where the species occur (Leibold 1995) and niche overlap among species (Pappas and Stoermer 1997), both recently suggested as useful tools for modeling the niches of invasive species (Mouillot et al. 2005; Di Febbraro et al. 2013). In addition, species distribution models (SDMs), which are numerical tools combining factors of the species presence or abundance with several environmental estimates, also depend strongly on niche characteristics (Soberón and Nakamura 2009). SDMs are increasingly being employed as predictive procedure in conservation planning and management of ecosystems (Elith and Leathwick 2009; Kosicki and Chylarecki 2014). Such models rely on the concept of the ecological niche occupied by the focal species and have traditionally been built and performed using species occurrence as the response variable and a range of environmental characteristics (land use composition or landscape metrics) as covariates (Guisan and Zimmermann 2000).

The modeling of species’ niches is of central importance in ecological applications. The accurate quantification of the niches can be useful in predicting species invasions, in the design of nature reserves and in forecasting possible effects of urbanization or climatic changes on species’ occurrence and ranges (Drake et al. 2006; Møller et al. 2011). This approach assumes that species distribute themselves based on niche spaces defined by climate and habitat features (Cardador et al. 2013).

However, the robustness of a model is influenced by several factors: the selection of relevant predictors and the modeling method, the interplay between all the environmental factors being considered, and the extent of extrapolation involved (Elith and Leathwick 2009). The goodness of fit of a statistical model (the measure of its performance or the amount of explained variance) describes how well it conforms with a set of observations (McCullagh and Nelder 1989). Any significant decrease in the performance of these models may result in negative implications for their application. Therefore, the identification of factors that may improve models performance is an important development (Smulders et al. 2010).

In order to define the niche of a species, multivariate statistical analyses, such as principal component analysis and redundancy analysis, are often used to create independent axes of resource utilization within a community (Doledec et al. 2000; Janžekovi and Novak 2012). Usually, the niche for each species is calculated from the product of the ranges of resource exploitations on each axis (Litvak and Hansell 1990). Typically, this process considers the various positive or negative relationships with environmental parameters (usually regarded as the abiotic factors) and the mostly negative relationships with other species (usually regarded as the biotic factors) such as competitive exclusion. Biotic interactions, however, affect species’ spatial patterns via several mechanisms including predation, competition, resource–consumer interactions, host–parasite interactions, mutualism, and facilitation, and these may also be positive in outcome (Bascompte 2009; Van Dam 2009).

**Figure 2:**
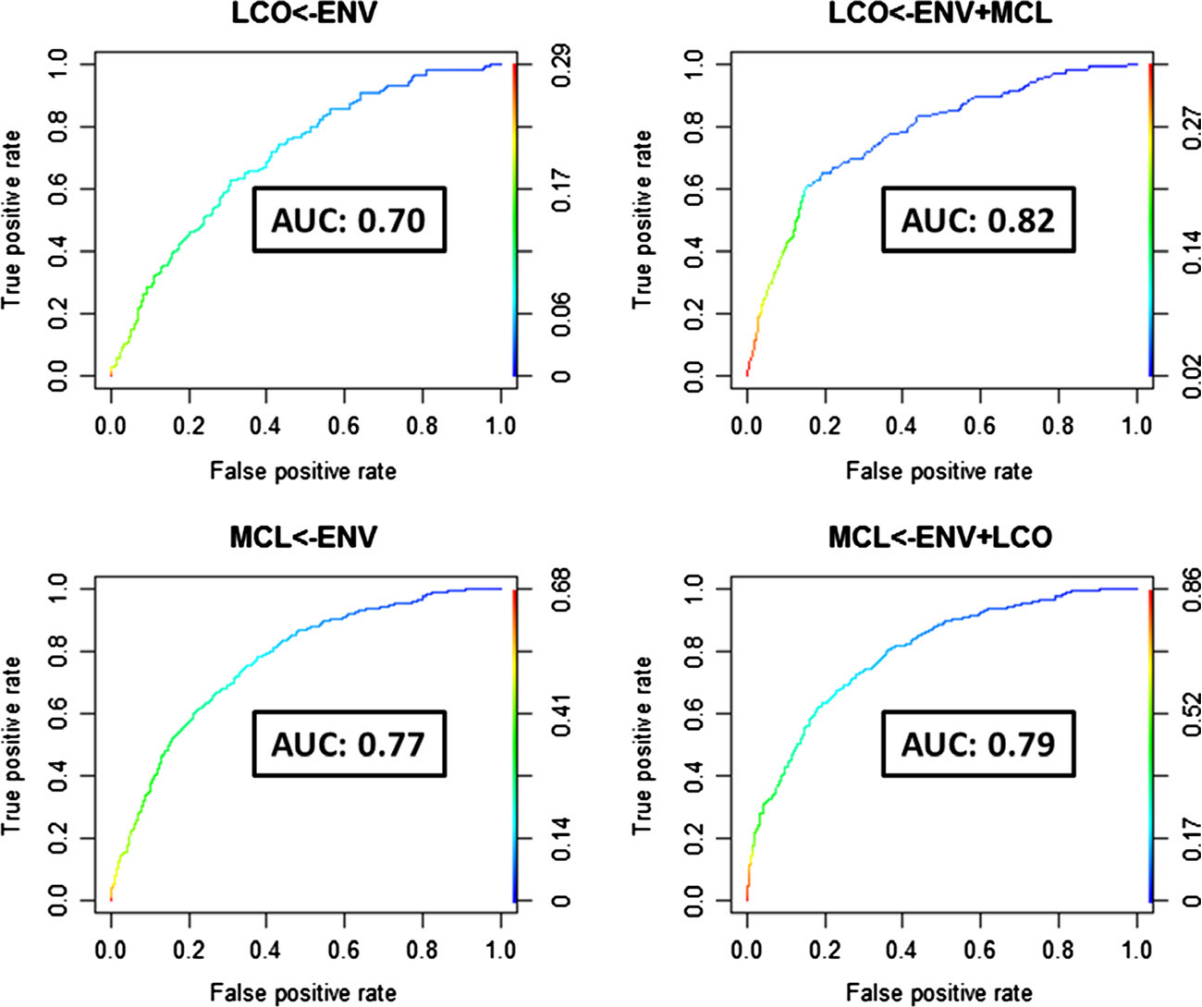
Evidence of gain on goodness of fit of species distribution models for two farmland bird species, studied in Central Italy, when were considered the heterospecific interactions (HTE) (Morelli and Tryjanowski 2014). Codes: the lines represent the area under the curve (AUC) of the best models performed first considering only the environmental variables (LCO<-ENV and MCL<-ENV) and after considering also the occurrence of associated bird species (LCO<-ENV+MCL and MCL<-ENV+LCO). LCO is *Lanius collurio*, MCL is *Milaria calandra*, and ENV are environmental parameters. The increase in performance of best model for red-backed shrike *Lanius collurio* when was considered the occurrence of corn bunting *Milaria calandra* was statistically significant.

In a recent study on species distribution models (SDMs) for bird species in agro-ecosystems in Central Italy, we found strong effects caused by bird species associations, and this significantly improved the goodness of fit of models (Morelli and Tryjanowski 2014). Our results demonstrated that adding ‘species-to-species’ interactions to baseline SDMs (performed on environmental variables at two spatial-scale level: land use and landscape scale) resulted in a significant improvement on models’ predictive power (see example in Fig. 2).

In this study, we tested the influence of ‘associated’ bird species on the avian niche occupation for red-backed shrike *Lanius collurio* in farmland of Western Poland, trying to quantify the potential improvement of predictive power of models when considering the interspecific interactions. In doing so, we aim to (1) summarize the main factors (biotic and abiotic) currently considered in ecology that can constrain the distribution of a species, (2) hypothesize about using the occurrence of heterospecific individuals, useful in detecting suitable habitat by some bird species, by employing ‘public information’, and (3) argue for the importance of considering biotic interactions in improving the SDMs process.

## Materials and Methods

### Study area, bird data collection, and environmental variables

The study was conducted in agricultural landscapes of Western Poland, near Odolanów (51.73 N; 17.49 E). The study area (38,000 ha) is an extensively used agricultural environment and comprises a mosaic of meadows and pastures (44%), arable fields (42%), midfield woodlots of different ages (6%), scattered trees, and discontinuous linear habitats, mainly mixed rows of trees and shrubs (see details in Hromada et al. 2002).

Sixty sites were sampled at least twice during the breeding season of 2010, between mid-April and the end of July. Bird species at sites were surveyed by 5-min point counts with each site being comprised of three points (each as a vertex of a triangle) at a distance of at least 300 m from one another at which expert observers counted birds from three independent directions. All counts were performed from half an hour after sunrise until 4.5 h after sunrise and only during favorable weather conditions without rain, snow, or strong wind. Point counts provide highly reliable estimates of relative population density and are a standardized and practical method for comparing bird communities between different habitats and times (Bibby et al. 1992; Voríšek et al. 2008). In order to verify the potential association between the occurrence of red-backed shrike and other bird species, we used a Pearson’s correlation matrix.

Environmental data were derived from descriptions of a 200-m radius area around the sampled point from the land cover map available for the region. The percentages of land uses within the buffer were calculated through ArcGIS 10 spatial analysis, using the following approach: (1) the creation of 200-m radius buffer areas around each sampled point, (2) employing an ‘intersect operator between buffer areas and land cover map, and (3) using a matrix cross-tab, to quantify the relative coverage of each land use classes. Land use categories were reclassified in larger groups, to obtain the following categories: building (which includes residential building, production facility, built with infrastructure, and processing areas), cultivated (which includes all cultivated and farmland categories), uncultivated, forest, reforest, grassland, shrubs, isolated trees, rivers, roads, and hedgerows. The environmental structures recorded were the presence and length (in meters) of power lines, hedgerows, and linear shrubs.

### Models procedure

The sites were confirmed as independent units as spatial autocorrelation values between geographic distance and land use dissimilarity among sites were low (Mantel test *r* < 0.10, *n* = 60, not significant) (Betts et al. 2006). Dissimilarity indices among sites were calculated using the “vegdist” function of the vegan package for R (Oksanen 2014), applying the Sørensen index of dissimilarity between pairwise sites for land use composition.

The nature and strength of relationships between red-backed shrike occurrence and environmental parameters or associated bird species were examined using generalized linear models (GLM) (McCullagh and Nelder 1989), with the dependent variable (bird occurrence) modeled by specifying a binomial distribution and environmental variables and associated birds used as predictors. Independent predictive variables were expressed as arcsin root square in the case of proportions. In order to avoid multicolinearity, parameters with the strongest correlation (>0.7) were manually eliminated. A stepwise backward procedure was followed in order to select the best predictors using AIC criterion (Akaike 1974).

The occurrence of red-backed shrike was modeled, first using environmental variables, and subsequently by adding the presence of cuckoo *Cuculus canorus*, a notably conspicuous species known to be associated with the occurrence of red-backed shrike, because one of its preferred hosts (Wesołowski and Mokwa 2013). Finally, the SDMs were performed adding the occurrence of three other species negatively correlated with red-backed shrike in the region: common Buzzard *Buteo buteo*, Eurasian Jay *Garrulus glandarius*, and hooded crow *Corvus cornix,* and with two species positively correlated, corn bunting *Milaria calandra* and common whitethroat *Sylvia communis*.

The comparison between model accuracy of the two approaches (classical approach performed only on environmental variables and approach using also biotic interactions) was run using the area under the curve of a receiver operating characteristic (ROC) plot and considering also the decrease/increase in number of predictors. The ROC is a graphical plot which illustrates the performance of a binary classifier system as its discrimination threshold is varied. It is created by plotting the fraction of true positives out of the positives (true-positive rate) versus the fraction of false positives out of the negatives (false-positive rate) at various threshold settings. The AUC calculated for each model indicates the predictive performance expressed as an index ranging from 0 to 1 (DeLong et al. 1988). The measure of the accuracy of AUC can be summarized as following: 0.90–1.00 excellent, 0.80–0.90 good, 0.70–0.80 fair, 0.60–0.70 poor, 0.50–0.60 fail (Swets 1988).

Graphically, the differing capacities of the models to predict species occurrence was explored by means of spatial patterns using interpolations with inverse distance weighted (IDW). The IDW interpolates a raster surface from several values of different points (probability of occurrence output from each SDMs in the sampled sites) and distances among points (Lu and Wong 2008). The spatial mismatch between patterns derived by SDMs and values obtained by actual occurrence of the species, collected in the field, indicates a lack of fit by the models.

All tests were performed using R (R Core Team 2014).

## Results

We obtained a total of 1046 records of 123 bird species during the breeding season of 2010 in farmlands of Western Poland. The average bird species richness was 14.89 (max: 23, min: 6). The red-backed shrike was present in more than 25% of sampled sites.

The occurrence of red-backed shrike was correlated positively with three bird species and negatively with other three species (Table 1). All these species were added as predictors for the model procedure which considered heterospecific interactions. However, only two of these were selected by stepwise automatic procedure, as predictors for the best model (cuckoo, positively correlated with red-backed shrike, and Eurasian jay, negatively correlated) (Table 2).

**Table 1:** Coefficients and p-values of Pearson’s correlation among the occurrence of red-backed shrike and selected bird species, in farmlands of Western Poland during the breeding season of 2010. Significant values are in bold

	*Lanius collurio*		*Cuculus canorus*		*Milaria calandra*		*Buteo buteo*		*Garrulus glandarius*		*Corvus cornix*		*Sylvia communis*	
	*r*	*P*	*r*	*P*	*r*	*P*	*r*	*P*	*r*	*P*	*r*	*P*	*r*	*P*
*L. collurio*	1	–	**0.29**	**0.01**	**0.18**	**0.05**	−**0.11**	**0.04**	**−0.29**	**0.01**	**−0.34**	**0.00**	**0.42**	**0.00**
*C. canorus*	**0.29**	**0.01**	1	–	0.03	0.83	0.03	0.83	0.01	0.92	−0.01	0.91	**0.51**	**0.00**
*M. calandra*	**0.18**	**0.05**	0.03	0.81	1	–	0.14	0.23	−0.17	0.16	0.14	0.25	0.06	0.63
*B. buteo*	−**0.11**	**0.04**	0.03	0.83	0.14	0.23	1	–	0.03	0.83	0.21	0.07	−0.06	0.60
*G. glandarius*	−**0.29**	**0.01**	0.01	0.92	−0.17	0.16	0.03	0.83	1	–	−0.08	0.50	−0.07	0.54
*C. cornix*	−**0.34**	**0.00**	−0.01	0.91	0.14	0.25	0.21	0.07	−0.08	0.50	1	–	−0.17	0.17
*S. communis*	**0.42**	**0.00**	**0.51**	**0.00**	0.06	0.63	−0.06	0.60	−0.07	0.54	−0.17	0.17	1	–

**Table 2:** Results of species distribution models (SDMs) for red-backed shrike in farmlands of Western Poland, modeled first on environmental variables, next on environmental variables plus one associated species, and finally on environmental variables plus six associated bird species. The table shows the parameter estimates, standard errors, *z*-values, and significances for the terms. Only significant parameters selected in the best models are showed

Models	Predictors	Estimate	SE	*z*	*P*
Model 1: on environmental variables	Forest	−6.07	4.46	−1.3	<0.05
	Shrub	16.78	8.05	0.9	<0.05
Model 2: on environmental variables plus *Cuculus canorus*	Forest	−10.26	4.59	−2.2	<0.05
	*C. canorus*	1.73	0.69	2.5	<0.05
Model 3: on environmental variables plus six associated bird species	Forest	−16.34	8.10	−2.0	<0.05
	Cultivated	−7.29	4.42	−1.6	<0.05
	Shrub	2.20	1.00	0.8	<0.05
	Building	−28.9	15.65	−1.9	<0.05
	*C. canorus*	3.33	1.35	2.5	<0.05
	*G. glandarius*	−5.44	2.00	−2.6	<0.05

**Figure 3:**
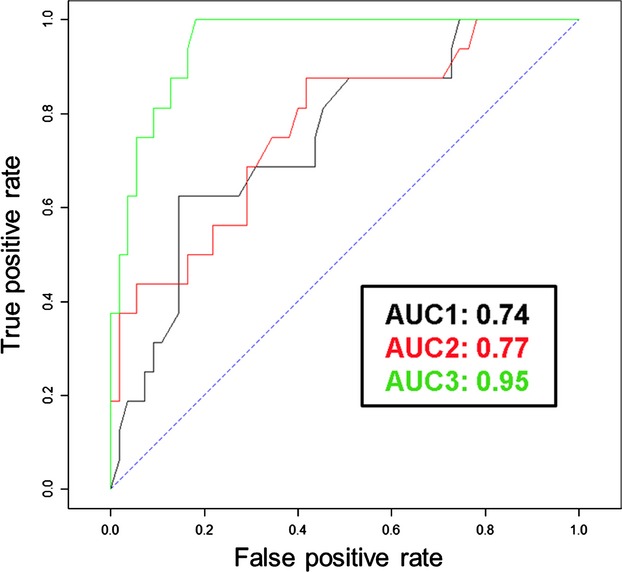
Evidence of gain on goodness of fit of species distribution models for the red-backed shrike *Lanius collurio*, studied in Western Poland, when were considered the heterospecific interactions (HTE). Codes: the lines in different colors represent the area under the curve (AUC) of the best models performed first considering only the environmental variables (black line), considering the environmental variables and also the occurrence of cuckoo *Cuculus canorus* as associated bird species (red line), and considering other bird species (green line).

An improvement in the accuracy of best SDMs for the red-backed shrike from 74 to 95% in correct classification was obtained (Fig. 3). The best model performed on classical environmental variables plus two associated bird species demonstrated “excellent” capacity to predict the occurrence of red-backed shrike (Fig. 3).

**Figure 4:**
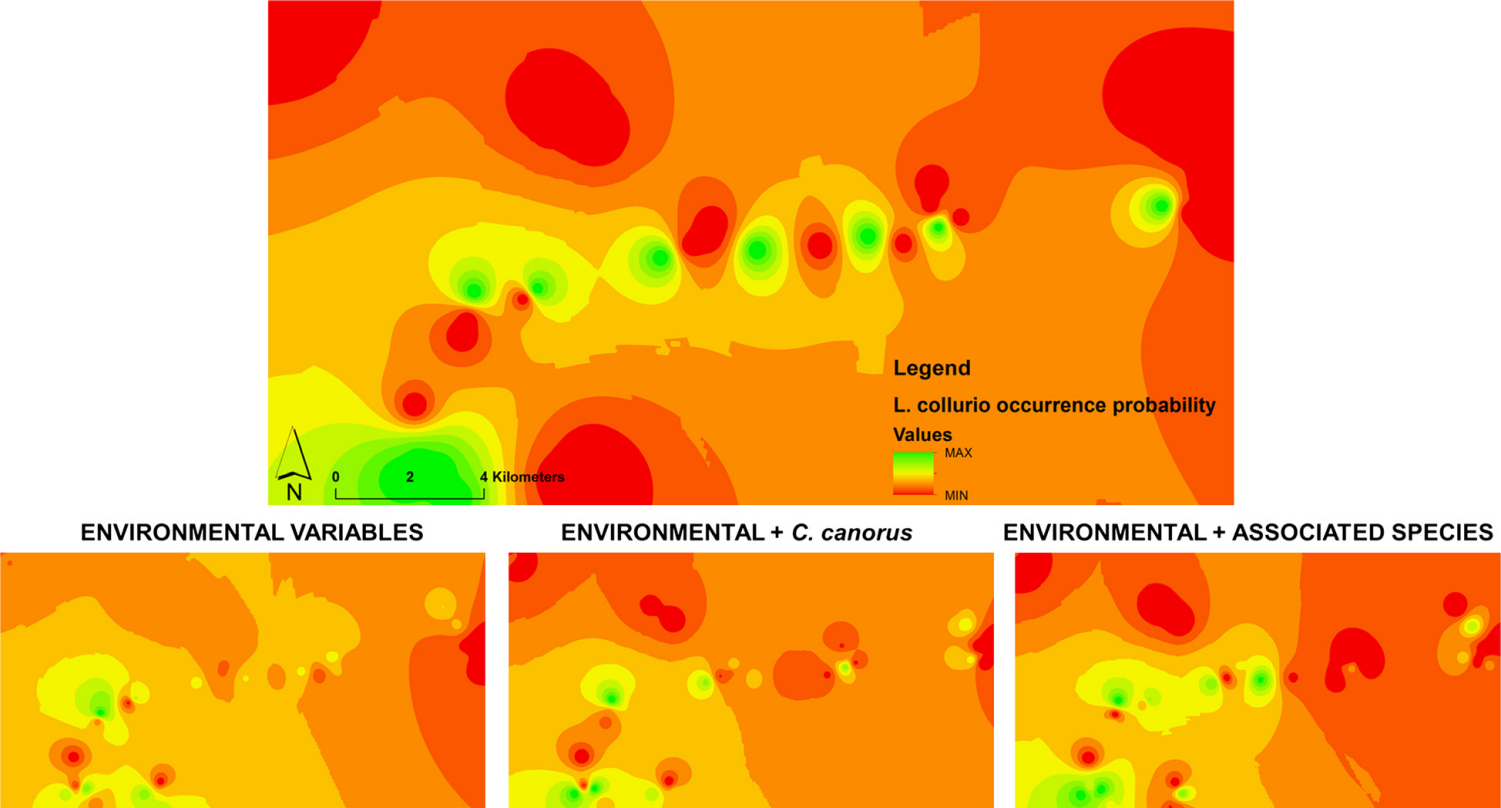
Inverse distance weighted (IDW) interpolation applied to study the spatial mismatch between the pattern of probabilities of occurrence of red-backed shrike predicted by the best SDMs with and without associated bird species (lower plates) and interpolation of probabilities based on the occurrence of species in the sampled sites, in Western Poland. The values of probability (ranged 0 to 1) are represented in a colored scale from red (lowest values) to green (highest values).

The Figure 4 shows how the model integrating environmental data plus biotic interactions fits with the actual field data of occurrence. Improved spatial congruence was obtained between best SDMs performed using both environmental variables and few associated bird species (lower right plate in the Fig. 4) and the real field data (upper plate in the Fig. 4).

## Discussion

### Biotic interactions and species distribution patterns

Our results confirmed similar findings obtained by another recent study (Morelli and Tryjanowski 2014), demonstrating the importance of including the occurrence of associated birds in the accurate identification of potential occupied niche using SDM inferences. Similarly, Ward et al. (2010) found that many bird species use both habitat (e.g. vegetation structure) and social cues (presence of conspecifics and/or heterospecifics) during the selection of a location for breeding. Moreover, several examples demonstrate how the presence of some species may positively affect the occupancy of a territory by other species (Wiklund 1979; Bogliani et al. 1999; Tryjanowski 2001; Hromada et al. 2008). As highlighted by Kissling et al. (2011), relatively little effort has been made to incorporate multispecies interactions at large spatial extents using interaction matrices, for example, in ‘species interaction distribution models’ (SIDMs). However, recent explicit considerations of the role of biotic interactions as information on habitat quality, protection against predators, etc. represent an important advance in the field of niche theory (Le Roux et al. 2013). Furthermore, for models performed at a macroscale, Heikkinen et al. (2007) have shown how the inclusion of biotic interactions (in this case, the distribution of woodpeckers as a predictor variable) significantly improved the explanatory power, cross-validation statistics, and the predictive accuracy of models for the distributions of several owls species.

We suggest that the set of measures currently used to quantify realized species niches could be improved significantly by the inclusion of data on the occurrence of associated species or species assemblages. The presence of such species may favor habitat selection, potentially constraining the supposed fundamental niche. This is a primary order affect, dependent by the presence of other species; it may not necessarily involve interactions such as competitiveness, resource partitioning, refuge visibility, or food availability of shared resources, but is an aspect of species assemblage. Such influences may be indirect, related to the availability of “public information” useful in assessments of habitat quality (Hromada et al. 2008; Morelli and Tryjanowski 2014) or antipredator awareness, processes with potentially strong consequences for the settlement of species (e.g. Tryjanowski 2001). From this perspective, we have assumed that an hypothetical species 1 may use the presence of a successfully established individual of species 2 as a form of indicator of habitat suitability. Although much information is acquired from members of the same species, important information can also be gathered from individuals of other species (heterospecifics). This may be particularly valuable in the case of heterospecifics belonging to the same trophic level, because these species often require similar resources and need to avoid similar predators. Theoretically, the cues and signals produced by other species are part of the “public information” available to many animals, through which animals may assess habitat, the presence of resources, or potential risks (Danchin et al. 2004; Valone 2007). As a consequence, the presence of heterospecifics may exert an importance influence on the settlement of breeding species in a territory (Ward et al. 2010).

**Figure 5:**
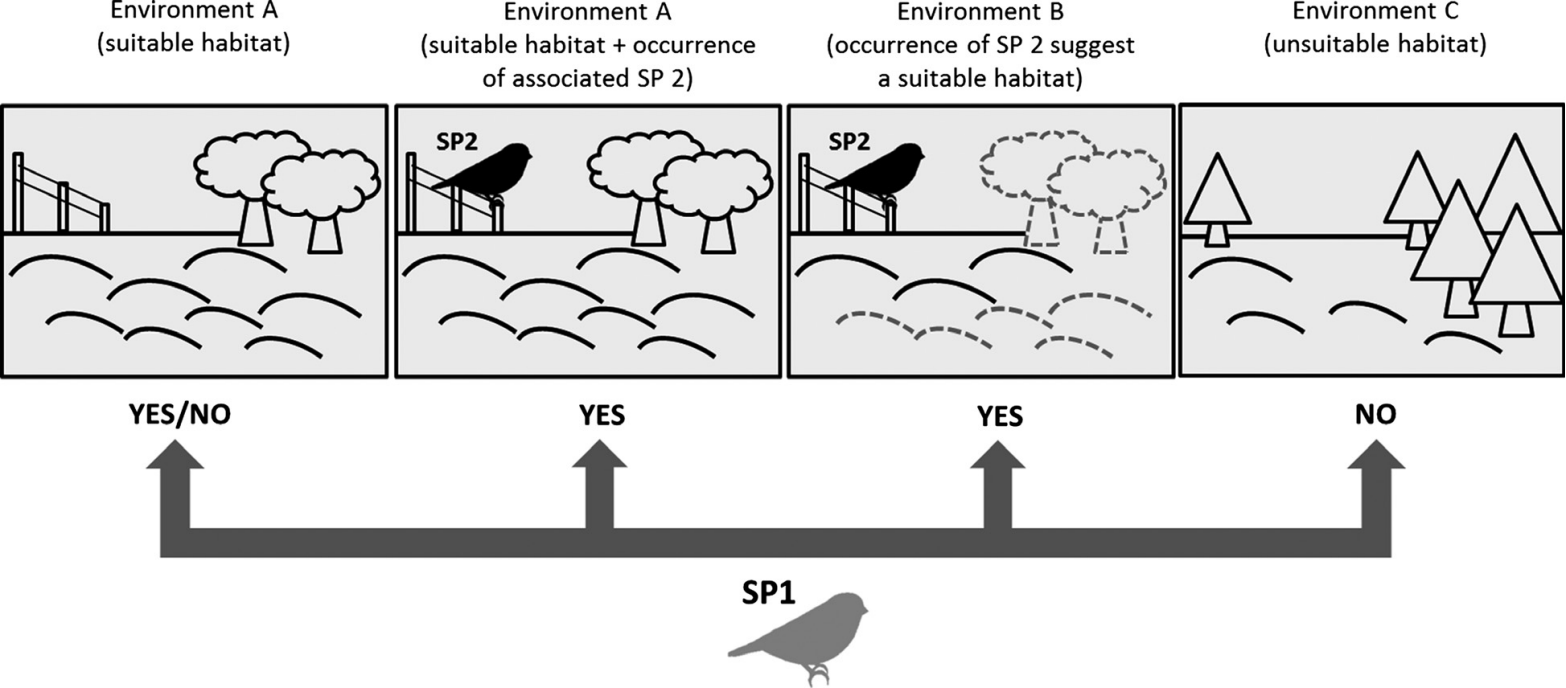
Simplified representation of the main mechanisms involved in the selection of the breeding territory by the hypothetical species 1 (SP1) and exemplification of the “heterospecific trace effect” (HTH) provided by the species 2 (SP2) to SP1 on the selection of a settlement territory in the environment B. The environments A and B are both suitable for SP1, while the environment C is unsuitable.

We propose that this kind of ecological process, the “heterospecific trace effect” (HTE), sensu stricto the presence of a species that can constitute a trace or indication (for another species), may influence decisions of whether to settle in a location. Such information would therefore be interpreted positively for the identification of a suitable habitat (Fig. 5). We use the label “trace” because such information would constitute an indirect signal, a factor not directly measurable (compared to many environmental characteristics) but suggested by the presence of another species. In fact, the use of public information appears to often depend on its cost of acquisition (Valone 2007), constituting an alternative way for animals to “read the environment.”

### Advantages and limitations on use of heterospecific interactions in niche theory and SDMs

Following Geange et al. (2011), descriptions of niche space often incorporate multiple axes, each of which may be an environmental condition (e.g. altitude, temperature, habitat openness), resource type (e.g. prey type, refuge type), a phenotypic trait, or an index of electivity (e.g. Manly’s Alpha). Multiple axes may be described by different types of data including binary data (e.g. presence/absence of determinate characteristic, or prey occurrence), categorical data (e.g. host size classes), continuous data (e.g. land use type coverage), count data (e.g. number of prey eaten per hour), or other indices (e.g. habitat use relative to availability). Within such a framework, the addition of a new axis with heterospecific interactions information is feasible, structured as binary data type (occurrence of associated species) as part of the suite of variables adequate to define the species’ niche. Information concerning the presence of potential associated species is relatively easily derived from a correlation matrix or by published data.

It is important, however, to distinguish between the implications of biotic interactions (e.g. HTE) for niche theory and that of models. Crucially, in order to analyze the association between two species in a modeling framework, the species occurrence used as predictor should be independent, as required by statistically necessity (Mac Nally 2000). For example, two species would be potentially suitable candidates if they settled into their territories at different times. An ideal case would be between one migratory species and one sedentary species, which become sympatric only during the breeding season. Other suitable candidates would be species out of phase in commencing breeding, or through differences in timing of spring arrival (Rolshausen et al. 2010; Morelli and Tryjanowski 2014) as well as those using structures or features built by other species, as for example, owl species that are often more frequent where woodpecker species also occur (Heikkinen et al. 2007) where the interactions relate to the availability of nesting cavities for secondary cavity nesters (Hakkarainen et al. 1997; Wesołowski 2007). In those cases, the use of the occurrence of associated species may be treated as a standard environmental variable such as those associated with the landscape or climate.

We can, nonetheless, envisage difficulties in discriminating between potential causes of spatial congruence for two species. For example, the shared niche space due to similar habitat requirements may overlap with our proposed heterospecific trace effect, by definition. However, we consider that heterospecific positive interactions (rather neglected in modeling) would be useful in some cases, such as when the only environmental description of a territory is not sufficient to explain the occurrence of determined species. On the other hand, the HTE should be a natural consequence of the “public information” theories, applied on SDMs.

**Figure 6:**
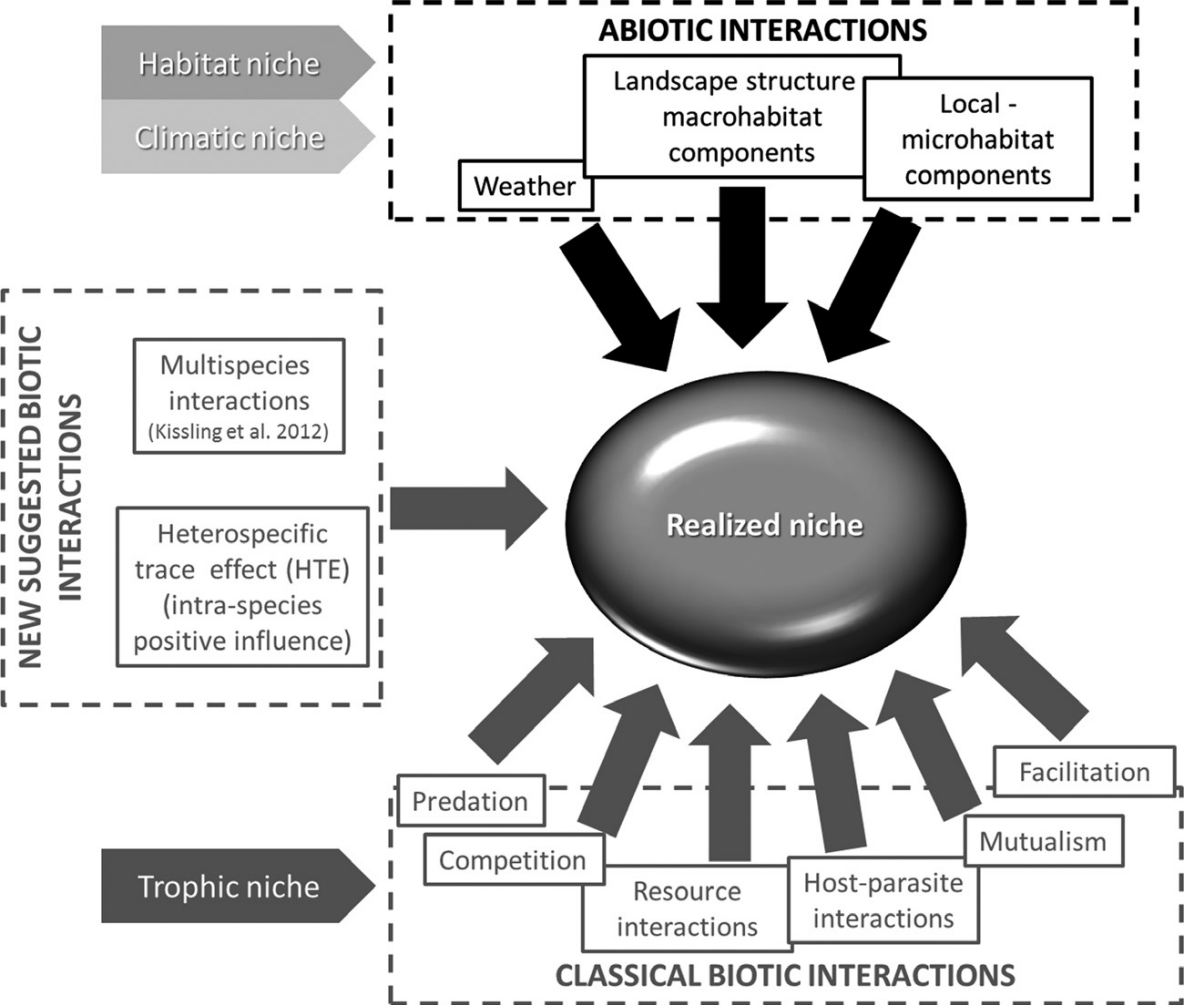
Schematic representation of main types of abiotic (black arrows) and biotic (gray arrows) interactions which define the niche of an hypothetical species. In the left side of the figure are highlighted the biotic interactions suggested in this article, in order to improve the accurate quantification of the realized niche of the species.

The network structure of interactions among species and their effects on population dynamics are keys to understanding the mechanisms by which biodiversity is maintained (Bascompte et al. 2003). In a recent review of community interactions under climate change, Gilman et al. (2010) argued that interactions among species can strongly influence how climate change affects species and that failure to incorporate these interactions limits our ability to predict species responses to alterations to the climate. One important step toward improving our capacity to predict future species assemblages will be to clarify the role of all potential biotic interactions (Wisz et al. 2013). Although the majority of classical models of species distribution are focused on the characteristics of single species and occupied environment or climatic envelope (e.g. Titeux et al. 2007; Kosicki and Chylarecki 2011, 2014; Kosicki et al. 2013), consideration of biotic interactions (e.g. the proposed HTE) could enhance the predictive capacities of SDMs. Biotic interactions can assist in explaining more completely the fundamental and the realized niche of species (Fig. 6). While it is clear that improving our understanding of the complexity of ecological models would enhance conservation objectives, previous studies have mainly focused on somewhat simplistic interaction types: antagonism, competition, or mutualism, despite the fact that various interactions coexist in nature (Mougi and Kondoh 2013). The implications of coexistence of the multiple interaction types on the maintenance of ecological community is an important issue, and numerous related questions have been left unanswered (Thébault and Fontaine 2010; Fontaine et al. 2011).

Other practical aspect related to the consideration of biotic interactions in SDMs includes the possibility of using associated species with high detectability (e.g. the cuckoo in the present study, e.g. Soler and Soler 2000) to study the potential distribution of other (associated) species with lower detectability rates. The use of more conspicuous species as surrogates of others should help to implementation of surveys strategies, potentially enabling ordinary citizens to take part of monitoring surveys (Jiguet et al. 2012). On the other hand, the HTE hypothesis suggests that available heterospecific information on settlement processes, as well as ecological factors, is required to explain the distributional patterns of some bird species. For all these reasons, we suggest an increase in the consideration of occurrence of potential associated species and the study of multispecies interactions (Kissling et al. 2011; Wisz et al. 2013) both in niche theory and in improving species distribution models.
